# Cognitive effects of brief and intensive neurofeedback treatment in schizophrenia: a single center pilot study

**DOI:** 10.3934/Neuroscience.2024021

**Published:** 2024-09-09

**Authors:** Fabrizio Turiaco, Fiammetta Iannuzzo, Giovanni Genovese, Clara Lombardo, Maria Catena Silvestri, Laura Celebre, Maria Rosaria Anna Muscatello, Antonio Bruno

**Affiliations:** 1 Department of Biomedical and Dental Sciences and Morphofunctional Imaging, University of Messina, Messina, Italy; 2 Psychiatry Unit, Polyclinic Hospital University of Messina, Messina, Italy; 3 Department “Scienze della Salute”, University of Catanzaro, Catanzaro, Italy; 4 Department of Mental Health and Addictions, ASST Papa Giovanni XXIII, Bergamo, Italy

**Keywords:** neurofeedback, schizophrenia, cognitive symptoms, working memory, apraxia

## Abstract

**Background:**

Schizophrenia is characterized by significant cognitive impairments and affects up to 98% of patients. Neurofeedback (NF) offers a means to modulate neural network function through cognitive processes such as learning and memorization, with documented structural changes in the brain, most notably an increase in grey matter volume in targeted regions.

**Methods:**

The present 2-week, open-label, preliminary study aims to evaluate the efficacy on cognition of an adjunctive short and intensive (8 daily sessions lasting 30 minutes) alpha/theta NF training in a sample of subjects affected by schizophrenia on stabilized treatment with atypical antipsychotic drugs. The efficacy was measured at baseline and at the end of the study by the Brief Neuropsychological Examination 2 (ENB 2), the Mini Mental State Examination (MMSE), and the Stroop color-word interference test; the clinical symptoms were assessed using the Positive and Negative Syndrome Scale (PANSS).

**Results:**

A final sample of nine patients completed the study. Regarding the cognitive performance, at the final assessment (week 2), the NF treatment significantly improved the performance in the “Story Recall Immediate” (p = 0.024), “Story Recall Delayed” (p = 0.007), “Interference Memory 30 s” (p = 0.024), “Clock Test” (p = 0.014) sub-tests, and the ENB2 Total Score (p = 0.007). Concerning the clinical symptoms, no significant changes were observed in the PANSS subscales and the PANSS Total score.

**Conclusions:**

NF could represent an adjunctive treatment strategy in the therapeutic toolbox for schizophrenia cognitive symptoms.

## 1. Introduction

Cognitive deficits are core features of schizophrenia, with up to 98% of patients exhibiting impairments in a wide range of cognitive functions such as working memory, attention, processing speed, and visual and verbal learning; such basic deficits reverberate on complex behaviors, and are subsequently expressed as substantial difficulties in reasoning, planning, abstract thinking, and problem-solving [1],[2]. Cognitive deficits primarily affect socio-relational and work functioning, with significant repercussions on the outcomes [3]; moreover, they still represent one of the most critical and challenging dimensions in the treatment of schizophrenia. It is well established that antipsychotic drugs are effective in treating positive symptoms; however, they do not address cognitive and executive dysfunctions [4], whose treatment essentially involves non-pharmacological interventions [5].

Neurofeedback (NF), a neuromodulation technique derived from biofeedback (i.e., a biofeedback to brain targets), allows patients to directly perceive brain activity as obtained by non-invasive devices, such as electroencephalography (EEG) or functional magnetic resonance imaging (fMRI), that are visually or auditorily represented in real-time through a brain-computer interface [6],[7]. Starting from the targeted visual or auditory representations of the brainwaves generated by neural activity, NF protocols aim to progressively enable subjects to consciously modulate and self-regulate their brain activity either through spontaneous, individual strategies, or learned models, such as operant conditioning, motor learning, dual process theory (feedforward and feedback), awareness theory, global workspace theory, and skill acquisition [6],[8],[9]. Thus, NF can be considered as a method to modify the function of neural networks through cognitive processes (learning and memorizing), and has been associated with structural changes in the brain, as documented by an increase in the grey matter volume of the targeted areas, probably mediated by processes of neuroplasticity and cortical growth [6],[8],[9]. Aimed at treating constellations of behaviors and functioning impairments arising from neural networks beyond specific symptomatic domains, NF has the potential to broaden our knowledge and understanding of the neurobiological foundations of the disorders.

The use of NF to improve cognitive performance in non-clinical samples is a recent area of research; available studies have shown that even intensive and short protocols that targeted alpha and theta waves were effective in improving working memory and attention, which are involved in executive functions [10]–[13].

NF has been used in several psychiatric conditions, such as depression, post-traumatic stress disorder (PTSD), attention deficit hyperactivity disorder (ADHD), and schizophrenia [14]. Many trials in samples of schizophrenia patients have employed NF to treat positive symptoms, mainly auditory hallucinations, using long-lasting standard protocols which have been rarely completed by this type of patient [15]–[19]. Few studies have applied long-lasting NF protocols to treat positive, negative, and cognitive symptoms at the same time [20],[21].

Regarding short protocols, fMRI-NF has been proven effective in reducing auditory verbal hallucinations by different mechanisms, including the following: the modulation of connectivity within the default-mode network, the downregulation of the superior temporal gyrus, and the control of the activity of the anterior cingulate cortex (ACC) [22],[23]. Other studies have evaluated the effectiveness of NF on emotional regulation and recognition in schizophrenia [24]. Less knowledge is available on NF as a potential treatment for cognitive impairment. A first speculation was proposed by Schneider et colleagues, who wondered if slow cortical potentials (SCPs) could reflect the regulation of attention resources in cortical neuronal networks, even though they did not assess the cognitive symptoms [25]. An EEG-NF training of 24 sessions in 12 weeks was effective to improve the working memory, as demonstrated by EEG markers of working memory (frontal P3 amplitude and gamma power) [26].

Based on this background, the present study aims to evaluate the effect of a brief and intensive NF treatment on cognitive deficits as the primary outcome and clinical symptoms as the secondary outcome in a sample of schizophrenia patients stabilized with atypical antipsychotics.

## 2. Methods

### 2.1. Study design

This was a 2-week, open-label, preliminary study aimed at evaluating the efficacy on cognition of an adjunctive short and intensive (8 daily sessions lasting 30 minutes) NF training, in addition to a stable atypical antipsychotic monotherapy. The antipsychotic dose was left unchanged, and no additional drugs (antidepressant/anticonvulsant/anxiolytic) were allowed during the trial period. Each neurofeedback session was carried out in a dedicated quiet space using the Encephalan Main ABP-26 with the Rehacor software (Medicom MTM Ltd., Taganrog, Russia). A screen-printed cap was placed on the subject's head to electrode positioning according to the 20-10 international system (Electrode 1: left central occipital shunt C3-O1; Electrode 2: C4-O2 right central occipital lead; Neutral electrode: on the subject's arm). To promote a better conductivity, an electroconductive gel was applied to the subjects' skin. Using a frequency band of 6 to 9 Hz, the patients were trained to increase the alpha and theta frequencies in the eyes-open condition: both visual (geometric forms, diagrams, graphics, linear distortions, images, slides, and videos) and auditory (audio fragments, sounds of nature, voice information, and noisy distortions) scenarios were provided. Moreover, audio and video feedback messages were presented to inform the participants of their training scores. Once the protocol was completed, the electrodes and cap were removed. Given the absence of studies evaluating the efficacy of short and intensive NF protocols on the cognitive symptoms of schizophrenia, the rationale for the choice of alpha and theta frequency bands was made according to studies conducted on healthy elderly subjects (Angelakis et al., 2007; Lecomte and Juhel, 2011; Reis et al., 2016).

The study was carried out at the Psychiatry Unit of the University Hospital of Messina, Italy, was conducted according to the Declaration of Helsinki, and was approved by the ethics committee of Messina (Prot. N. 32/19, April 15, 2019).

### 2.2. Subjects

The accidental sampling method was used to recruit the participants within the study. Ten outpatients, 6 men and 4 women, aged between 18 and 55 years (mean age ± SD, 36 ± 2.3 years), affected by schizophrenia according to DSM5 criteria, and in stable atypical antipsychotic (clozapine, olanzapine, risperidone, and paliperidone) monotherapy at least 3 months were included in the study. The exclusion criteria included a comorbidity with any other major psychiatric disorder, significant concomitant medical pathologies, organic brain disorders, a current diagnosis or history of alcohol or substance dependence (excluding nicotine), dementia, and an intellectual disability. All patients who participated in the study regularly provided written informed consent after a comprehensive and detailed explanation of the research protocol.

### 2.3. Instruments

During the study, patients underwent two visits: baseline (day 0) and final (week 2). The efficacy was evaluated using objective tests that assessed multiple domains of cognitive and executive functioning:

• the Brief Neuropsychological Examination 2 (ENB 2) [27] is an assessment battery validated on the Italian population from 15 to 96 years that offers a qualitative and quantitative analysis of patient cognitive performance. The battery consists of 16 tests (Digit Span, Story Recall, Immediate Story Recall, Delayed Interference Memory 10s, Interference Memory 30s, Trail Making parts A and B, Token, Phonemic Fluency, Abstract Reasoning, Cognitive Estimation, Overlapping Figure, Spontaneous Drawing, Copy Drawing, Clock Drawing, and Apraxia) and a Total score that analyzes the following cognitive areas: short, long term, and working memory, integration capacity, visual-spatial research, divided attention and attention shifting/switching, psychomotor speed, verbal comprehension, lexical recovery, logical and abstract reasoning, critical sense, capacity for discrimination, complex copy skill, praxis, and mental representation. For each ENB-2 test, the scores are presented as mean ± standard deviation (SD) and a global normative value.

• the Mini Mental State Examination (MMSE) [28] is a 30-question measure that tests five areas of cognitive function: orientation, registration, attention and calculation, recall, and language. The presence of cognitive decline is determined by the total score. The maximum score is 30. Traditionally, a 23/24 cut-off is used to select patients with a suspected cognitive impairment.

• the Stroop color-word interference test [29] is a neuropsychological test used to assess the ability to inhibit cognitive interference. It consists of 3 trials to be executed as fast as possible: 1) read the names of colors printed in black ink (W); (2) name different color patches (C); and (3) name the color of the ink instead of reading the word (CW). The Stroop challenge is measured as reaction times, and the number of correct responses made in 120 seconds is recorded. The difference between the neutral and conflict conditions is often taken as a measure of interference.

The clinical symptoms were evaluated by the Positive and Negative Syndrome Scale (PANSS) [30]. Any observed and/or spontaneously reported adverse effects were obtained by nonspecific querying and classified in terms of the onset, duration, severity, action taken, and outcome. Trained raters with at least 3 years of clinical experience in each measure administered the data for the assessments according to standardized administration and scoring procedures; each patient had the same person administer the psychopathological and cognitive tests and conduct the clinical interviews.

### 2.4. Statistical analysis

As this was a pilot study, no formal sample size calculation was performed. Because of the small sample size, the analyses were carried out by nonparametric tests. A “per-protocol” (PP) analysis was performed, and the outcome analyses were conducted only on the subjects who completed the study. The continuous data were expressed as mean ± Standard Deviation (SD), and the within-group differences between the baseline and final time of the study were assessed by the Wilcoxon rank sum test. In addition, a Cohen'd statistic was applied to measure the magnitude of the treatment effect: the effect size was considered small when it was lower than 0.50, moderate when it ranged from 0.50 to 0.79, and large when it was 0.80 or greater. The results for p-values <.05 were considered significant; moreover, considering that multiple correlations increase the risk of Type 1 errors, a Bonferroni correction was performed.

## 3. Results

Nine patients completed the study (90% completion rate); the only drop-out was due to non-compliance. The clinical-demographic characteristics of the sample are reported in Table 1.

**Table 1.** Clinical and demographic features.

Clinical-demographic characteristics
Patients enrolled/Patients completers	10/9
Gender (M/F)	6/4
Age (years), mean ± SD	36±2.29
Level of education (years), mean ± SD	9.67±2.50
Antipsychotic	**N**
Clozapine	2
Olanzapine	3
Risperidone	3
Paliperidone	2

Table 2 shows the baseline (week 0) and final scores (week 2) on the cognitive assessments and the relative treatment effect size. Regarding the cognitive performance, NF treatment significantly improved the performance in the “Story Recall Immediate” (p = 0.024), “Story Recall Delayed” (p = 0.007), “Interference Memory 30 s” (p = 0.024), “Clock Test” (p = 0.014) sub-tests, and the ENB2 Total Score (p = 0.007) at the final assessment (week 2). There were no significant differences in the other cognitive parameters assessed, which remained substantially unchanged compared to the beginning of the study, although there was a trend towards a general improvement except for the “Trail Making Test B” subtest, which slightly worsened compared to the baseline. After the Bonferroni correction, no significant differences between the baseline and Week 2 emerged. At the end of the study, the effect of the treatment was large in the ENB2 subtests “Story Recall, Delayed”, “Interference Memory 30s”, “Spontaneous Drawing”, “Clock Drawing”, “Apraxia” and in the MMSE, medium in “Story Recall, Immediate”, “Interference Memory 10s”, “Cognitive Estimation” and “Total score”, and small in all other cognitive explored dimensions.

**Table 2.** Cognitive Function Changes and Effect Sizes for Efficacy Measures in Patients Receiving NFB at Baseline and Week 2.

	Baseline (T0)		Week 2 (T1)		Wilcoxon Test p	Cohen's d d
	Mean	SD	Mean	SD		
ENB 2						
Digit Span	7.00	.87	7.00	.87	1.00	0
Story Recall, Immediate	10.67	4.36	12.67	2.65	.024	.50
Story Recall, Delayed	11.67	2.78	15.67	2.50	.007	1.50
Interference Memory 10s	6.33	1.32	7.33	2.50	.369	.50
Interference Memory 30s	5.00	3.12	8.00	.87	.024	1.30
Trail Making Part A	34.00	13.75	34.33	13.43	.335	0
Trail Making Part B	94.67	29.01	117.67	65.59	.857	.40
Token	5.00	.00	5.00	.00	1.00	0
Phonemic Fluency	13.33	6.56	13.67	7.05	.713	0
Abstract Reasoning	4.67	2.00	4.67	2.00	1.00	0
Cognitive Estimation	4.33	.50	4.67	.50	.083	.70
Overlapping Figure	29.00	14.31	31.00	13.75	.369	.10
Spontaneous Drawing	1.67	.50	2.00	.00	.083	.90
Copy Drawing	2.00	.00	2.00	.00	1.00	0
Clock Drawing	9.16	.66	9.83	.25	.014	1.30
Apraxia	5.33	1.00	6.00	.00	.083	.90
Total score	74.33	10.58	80.33	8.32	.007	.60
MMSE	28.67	2.00	30.00	.00	.083	.90
Stroop test	52.67	58.00	36.67	25.10	.369	.30

Concerning the clinical symptoms, no significant changes were observed in the PANSS subscales and the total score, although a trend towards an improvement in the symptoms can also be observed. At the end of the study, the treatment effect was small in all clinical PANSS domains (Table 3).

**Table 3.** Clinical scores and Effect Sizes for Efficacy Measures in Patients Receiving NFB at Baseline and Week 2.

	Baseline (T0)		Week 2 (T1)		Wilcoxon Test p	Cohen's d d
PANSS	Mean	SD	Mean	SD		
Positive	8.33	1.32	8.00	1.50	.83	.20
Negative	9.33	1.80	8.67	1.80	.83	.40
General Psychopatology	21.00	3.77	19.33	3.04	.83	.40
Total score	38.67	6.56	36.00	6.24	.83	.40

Finally, the intensive NF training was well tolerated, as demonstrated by the good compliance of the patients who participated in the study; in addition, as expected, no adverse effects or undesirable changes in the clinical parameters were reported or observed.

## 4. Discussion

Neuromodulatory techniques and direct brain training, which are supported by the ongoing advances in computational and engineering sciences, have reached the current status of potential promises for a non-invasive approach to psychiatric disorders; furthermore, such treatments can be personalized to target symptoms or functional domains within a dimensional framework that potentially addresses the neurobiological substrates of mental illnesses.

To the best of our knowledge, this is the first clinical trial aimed at evaluating the efficacy of brief and intensive treatments with NF on cognitive symptomatology in a sample of schizophrenia patients on stabilized treatment with atypical antipsychotics.

The obtained results showed that the short and intensive NF protocol resulted in a significant improvement in long-term (“Immediate” and “Deferred” Prose Memory) and working memory (Interference Memory), apraxia, abstract thinking, and planning (Clock Test) after eight sessions; in addition, an improvement in the global cognitive efficiency was observed, as evidenced by the ENB2 Total Score. Although not significant, a trend toward improvement was observed in other examined cognitive domains, such as verbal memory, selective attention, the ability to execute verbal commands, lexical access and retrieval skills, logical reasoning and abstraction skills, cognitive estimation, and visual recognition skills. Conversely, the dimensions of cognitive flexibility and shifting skills, as measured by the Trail Making Test B, resulted in slightly worsened measurements, although not statistically significant, at the end of the treatment. Our results are barely comparable with existing findings in the literature, as only sparse studies to date have evaluated the effect of NF on cognitive/executive functioning in patients with schizophrenia. The obtained data are almost congruent with findings from the general populations [10]–[13] and from two case reports that showed improvements in the reaction time, alertness and selective attention under go/no-go conditions after NF treatment for negative symptoms [31]. The efficacy of a short and intensive NF protocol (4 consecutive days with a total training duration of 13.5 hours) on clinical symptoms of schizophrenia has been evaluated in a case report in which the treatment was aimed at increasing the relative amplitude of the alpha/beta2 ratio (20–30 Hz) at the right parietal level (P4); beyond positive and negative symptoms, both the short-term memory and language patterns improved both immediately and in the 22-month follow-up [32]. Finally, as reported in a letter to the Editor, hemoencephalography neurofeedback (HEG-NFBK) performed in 10 sessions of 1 h twice a week led to improvements in nearly all the examined cognitive domains, including information processing speed, attention processing, working memory, executive functioning, and verbal and visual learning [33]. Thus, considering that the existing literature evidence for cognitive rehabilitation programs in schizophrenia are modest and uncertain regarding the efficacy and durability (McCutcheon et al., 2023), NF can represent a safe and easy-to-administer treatment to improve the cognition and daily functioning in these patients.

Furthermore, no significant changes were observed in the clinical symptoms, although a trend towards improvements in all the PANSS subscales and the PANSS Total score can also be observed. Even though they are not statistically significant due to the small sample size, these results are congruent with previous studies that have employed standard NF training to treat positive and negative symptoms in schizophrenia patients, which stated that NF can be effectively used as an add-on therapy in schizophrenia rehabilitation programs (Markiewicz et al., 2021; Singh et al., 2020).

Finally, the intensive NF training was safe and well tolerated, as stated by the low dropout rate in the sample during the treatment period and the absence of adverse events.

Although encouraging, our results should be interpreted with caution due to several limitations, which are mainly represented by the small sample size, the short-term follow-up, and the open design of the study, which limit the generalizability of the findings, and do not allow to exclude the presence of practical and placebo effects in the cognitive assessments. Additionally, no follow-up information was available, and it is not known whether the cognitive improvements were maintained over time.

Beyond limitations, an intensive NF alpha/theta protocol, if effective, may overcome one of the main problems encountered in the treatment of schizophrenia, either pharmacological or not, namely adherence to treatments and, more generally, reduced compliance.

## 5. Conclusions

NF could represent an adjunctive treatment strategy in the therapeutic toolbox for schizophrenia, alongside a consideration for the variety and flexibility of treatment protocols based on different brainwaves, and/or targeting different brain areas, circuits, and neural networks, thus realizing a form of personalized, precision treatment for symptomatic, cognitive, and functional domains. However, to date, most clinical trials which aimed to test the NF efficacy in schizophrenia were small in number and scale (case reports, case series); therefore, further studies with adequately powered and well-designed methodologies (randomized, controlled with sham NF protocol, long-term follow-up clinical trials) are needed to better evaluate the effectiveness of NF treatments on cognitive deficits and other symptomatic dimensions in schizophrenia.
